# A hybrid approach for accurate skin lesion segmentation using LEDNet and Swin-UMamba

**DOI:** 10.1038/s41598-026-38056-y

**Published:** 2026-02-06

**Authors:** Muhammad Ahtsam Naeem, Shangming Yang, Muhammad Asim Saleem, Ashir Javeed, Tanveer Ahmad

**Affiliations:** 1 School of Information and Software Engineering, University of Electronic Science and Technology of China, Chengdu, China; 2 Department of Electrical Engineering, Center of Excellence in Artificial Intelligence, Machine Learning, and Smart Grid Technology, Faculty of Engineering, Chulalongkorn University, Bangkok, 10330 Thailand; 3 Department of Computer Science, Blekinge Institute of Technology, Blekinge, Sweden; 4 Department of Computer Science, University of Cyprus, CYENS Centre of Excellence Nicosia, Nicosia, Cyprus

**Keywords:** Skin lesion segmentation, Edge detection, Medical imaging analysis, Deep learning, Multi-scale segmentation, Computational neuroscience, Data processing, Machine learning, Statistical methods

## Abstract

Accurate delineation of skin lesions in images is important for skin cancer detection. Existing methods often struggle with inherent complexities, such as irregular boundaries, textures, and artefacts in skin lesions. The study proposes a hybrid model comprising the edge-accurate LEDNet and Swin-UMamba for multiscale segmentation. The irregular boundaries and complex textures of skin lesions can be captured more effectively through this integration than with previous stand-alone methods. The structure of LEDNet includes components that enable it to segment lesions of various types effectively. Swin-Mamba is an encoder that uses Mamba-based architecture with the additional component of the VSS block. The proposed model is evaluated on the Ph$^2$, ISIC-2017 and ISIC-2018 skin cancer datasets and demonstrates robust performance across all datasets. The method achieved a Dice Similarity Coefficient (DSC) of 0.9734, a sensitivity of 0.9697, a specificity of 0.9858 and an accuracy of 0.9847 with ISIC 2017, DSC of 0.9753, a sensitivity of 0.9494, a specificity of 0.9902 and an accuracy of 0.9713 with ISIC 2018; and a DSC of 0.9801, a sensitivity of 0.9892, a specificity of 0.9966 and an accuracy of 0.9932 with Ph. These results show that the proposed hybrid framework has the potential to bring important benefits in the segmentation of skin lesions and is promising in clinical dermatology.

## Introduction

Skin cancer, especially melanoma, is one of the deadliest cancers due to its rapid progression and high metastatic potential and contributes significantly to global skin cancer-related mortality^1,2^. Due to advances in treatment methods, early diagnosis and accurate segmentation of skin lesions have become crucial^3^. In medical imaging applications, deep learning and computer vision technologies can be used for automated diagnosis and segmentation of skin and brain images with relevant performance and scalability^4^. Dermoscopy is a non-invasive imaging technique. It enhanced diagnostic accuracy through observing skin microstructures in high resolution^5^. However, dermoscopic image interpretation is a subjective and labour-intensive task, so disagreement may arise depending on the clinician^6^. To address these limitations, recent advances in deep learning have led to the development of automated segmentation systems that provide accurate, reproducible and highly precise analysis of dermoscopic images^7^ to assist dermatologists in clinical decision-making. The MaxViT modifications, which incorporate ConvNeXtV2 and GRN-based MLPs, demonstrated excellent capability and efficiency with small and diverse image datasets for cervical cancer diagnosis^8^.

In the segmentation of skin lesions, transformer-based methods such as TransUNet^9^ and SegFormer^10^ have proven to be promising, as they contain introspection mechanisms for modelling global features. However, these approaches often lack accuracy in delineating boundaries, which is essential for dermoscopic image segmentation. Our proposed solution is a hybrid architecture based on the edge-aware LEDNet^11^ for improved spatial precision and the Swin-UMamba transformer for global context through dynamic token representation. This combination enables detailed boundary demarcation while supporting the understanding of lesion structures across diverse lesion types. Many segmentation methods, including convolutional neural network (CNN)-based models, still experience performance issues at the boundaries because some spatial information is lost in the deeper layers of the network. Although convolutional neural networks (CNNs) and their derivatives, such as the U-Net architecture, are essential in biomedical image segmentation^12^, these models can blur object boundaries due to downsampling^13^.

To overcome these issues, modern models have employed strategies such as hybrid architectures and deep feature fusion techniques^14^. For example, the integration of state space models (SSMs) into CNNs has been shown to improve the accuracy of boundary detection and segmentation by modelling long-range dependencies within the image data^15^. Based on these recent advances, we propose an edge-guided UNet Mamba model that introduces an edge control mechanism into the UNet Mamba architecture. The reduction of the CNN blocks with the SSM components improves the accuracy of the boundary, leading to more accurate and reliable predictions and a sharper definition of the boundary.

The proposed Lesion Edge Detection Network (LEDNet) and Swin-UMamba model were tested on commonly used Ph$^2$, ISIC 2017 and ISIC 2018 datasets. The ISIC 2017 and ISIC 2018 produced by the International Skin Imaging Collaboration (ISIC) have proven to be state-of-the-art datasets in the field of dermoscopic image segmentation and can be used for comparative research studies due to the extensive annotated dermoscopic image sets they contain^16^. The Ph$^2$ database is useful for assessing the accuracy of segmentation across multiple lesion types. A study of an automated melanoma detection system built using the UNet Mamba framework, which applies an edge-driven scheme, demonstrates a promising advantage in improved segmentation and resolution. The methodology described below consists of several steps.

1. A hybrid model combining Swin-UMamba and LEDNet models for multilevel segmentation and edge detection is proposed to increase the accuracy of lesion detection.
2. LEDNet uses a Siamese architecture, a difference analysis module (DAM) and an edge guidance module (EGM) to capture complex lesion boundaries and improve segmentation accuracy.
3. The proposed model performs better on key metrics (e.g., Dice Similarity Coefficient, Sensitivity and Accuracy) than traditional methods such as U-Net and SCR-Net.
4. The model demonstrates strong potential for real-world clinical applications and provides reliable segmentation of skin lesions for early skin cancer detection.

### Study distribution

The remaining sections are organized as follows: Section “Related work” discusses related studies. Section “Methodology” is the methodology. Section “Evaluation metrics” discusses the evaluation metrics, Section "Experimental results and discussion" discusses the experimental results, Section “Discussion” discusses the discussion and Section "Conclusion and future work" discusses conclusions and future work.

## Related work

Deep learning models have been widely studied and analyzed in previous research to improve segmentation accuracy in skin lesion analysis^17^, innovative architectures and hybrid approaches. Among these, the U-Net architecture^18^, originally designed for biomedical segmentation, has been widely adopted for skin lesion segmentation due to its encoder–decoder structure, which effectively captures both local and global features. However, conventional U-Net models face difficulties in maintaining spatial integrity in fine details, often resulting in blurred or incomplete lesion boundaries^19^. To overcome these limitations, several modifications have been proposed, including multilevel feature extraction, residual connections and transfer learning strategies to improve the accuracy of the delineation and^20^. As a result, automated segmentation is one of the essential methodologies for enhancing the accuracy, speed and reliability of medical image diagnosis, enabling effective and timely decision-making^21–23^. Advances in deep neural network architectures, as demonstrated in recent ImageNet competitions, have improved performance on medical image segmentation benchmarks^24–26^. For accurate segmentation, it is necessary not only to mark the regions of interest but also to associate local image details with global contextual relationships^27^. To solve this problem, VMamba^28^ presents a vision backbone based on Mamba that builds hierarchical representations.

Research indicates that ViT-based implementations outperform CNNs in diagnostic tasks. Specifically, using combined Pap smear datasets, ViT-based methods achieved 99.48% accuracy in screening for cervical cancer^29^. Guidance methods along edges are particularly helpful for skin lesion segmentation, given the importance of precisely delineating the boundaries between benign and malignant lesions. The Fuzzy U-Net model employs a fuzzy logic mechanism, such as a May Fly Optimizer, to enhance the parameters of the U-Net model for optimised edge detection. This refines boundary definition and reduces noise. This approach is significantly better than standard CNNs^30^. The Res-UNet model combines the functions of the U-Net and ResNet models to achieve excellent lesion segmentation performance on the ISIC-2017 and Ph2 datasets^31^.

Lightweight hybrid architectures that combine convolutional backbones with attention mechanisms for skin cancer analysis have also been a focus of recent research. These models improve generalisation in imbalanced datasets and are applicable to devices with limited resources, such as mobile platforms^32^. The combination of CNNs and other methods in hybrid models has also shown its effectiveness in overcoming the difficulty of high spatial variations and low contrast values in dermoscopic images^33^. Such models are the RetinaNet-Mask, R-CNN-Hybrid-CNN discussed in^34^, which utilizes the advantages of a multi-level feature hierarchy. This enables accurate lesion delineation and effective differentiation between lesions and non-lesions, even when images are affected by occlusions and artifacts. These advancements highlight the potential benefits of incorporating edge guidance mechanisms within CNN-based frameworks^35^. In addition, the transformer modifications were promising in skin imaging. HSW-MSA Swin transformer structural designs using SwiGLU as layers have shown significant results in classification by effectively modelling the long-range dependencies and accelerating computational performance^36^.

Deep Supervised Multiscale Representation (DSM) addresses the problem of feature extraction at multiple levels. It is a multiscale connectivity-based model trained on the ISIC 2017 dataset and uses a CRF-based post-processing step to sharpen the lesion masks. These features significantly improve segmentation accuracy and robustness for complex cases^37^. Transfer learning with a modified U-Net and LinkNet architecture was used and significant segmentation results were obtained in the Ph$^2$ and DermIS datasets in^38^. The ISIC 2017 and ISIC 2018 challenges have already become the standard of dermoscopic image analysis^39^, which is a difficult criterion for segmentation accuracy and evaluation strategies. Participants who have participated in such competitions have developed segmentation models that focus on accurate lesion boundary detection, feasible feature extraction and performance on different types of lesions. The use of CNN-based architectures has improved significantly, with advances in CRF-enhanced networks and deep hybrid CNNs with edge-sensitive data^40^.

The Mamba architecture, originally used for long sequential tasks, has recently been modified for image processing tasks. It combines state space models (SSM) with CNN architectures and demonstrate better computational complexity and preserves spatial information. Through these advances, the proposed edge-guided UNet Mamba uses the idea of the edge-guided approach to improve boundary segmentation, especially in the difficult cases of dermoscopic images that need to be detected and segmented with low contrast and artifact-mitigated complex edges^41^. This study addresses the urgent need for improved robustness and accuracy in automated skin cancer diagnosis using the Ph$^2$, ISIC-2017 and ISIC-2018 datasets.

## Methodology

In this study, we used two models, LEDNet (Lesion Edge Detection Network) and Swin-UMamba, to extract lesion edges and segment these lesions using three skin cancer image datasets Ph$^2$, ISIC-2017 and ISIC-2018. The proposed framework combines an LEDNet architecture that is lightweight, an edge-sensitive decoder and Swin-UMamba, an encoder designed to leverage the strengths of CNN-based detail extraction with transformer-based semantic inference.

The dual-branch design enables more accurate edge detection compared to pure transformers. In particular, LEDNet accurately detects the edges, while Swin-UMamba uses these edge representations in its segmentation flow to achieve good segmentation of the lesions. To minimize the risk of overfitting, we applied the following strategies. We removed weights to ensure that the extremes of the data distributions in the cellular alphabet are not overemphasized in the resulting arc labelling and analyzed the per-cell validation to ensure that we did not overfit the problem and also implemented additional edge map features that focus more on structural patterns than noise. To solve the underfitting problem, edge maps and RGB channels were added to the input features and the complexity of the models was reduced by properly tuning the hyperparameters, choosing a loss function and symmetrically splitting the data set. The two-level modular structure demonstrates the efficiency of combining specific models, which enables correct segmentation and generalization of lesions.

**Fig. 1:**
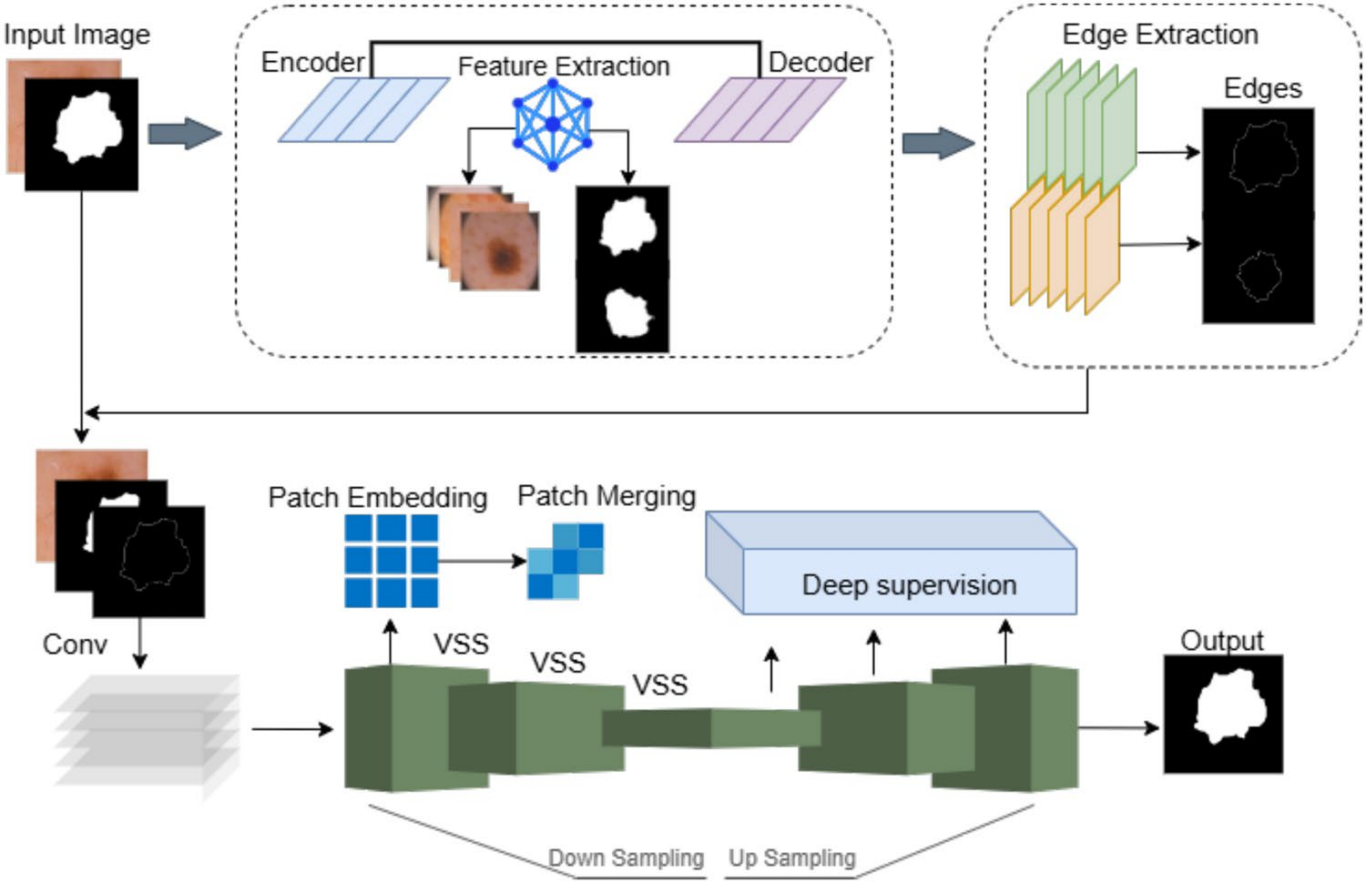
Framework of the proposed model.

The hybrid architecture is a combination in which the strengths of LEDNet and Swin-Mamba are utilized. The fine edge detection in LEDNet is achieved by edge-aware modules, enabling the preservation of spatial details and precisely delineating irregular lesion edges. Swin-UMamba has improved this by enhancing semantic interpretation through extensive dependency modelling and feature representation through hierarchy. Overall, the framework localizes fine-grained lesion boundaries and, at the same time, captures the global context, overcoming the limitations of purely spatial and purely semantic approaches. The overall framework is illustrated in Fig. 1.

### Edge extraction using Lesion Edge Detection Network (LEDNet)

First, the Lesion Edge Detection Network (LEDNet) is used to improve the accuracy of lesion boundaries. The proposed model uses the prior information about edge configurations to model complex and unclear boundaries of skin cancer lesions. LEDNet was developed on the basis of a Siamese network designed for the detection of multi-level features from paired images. During feature extraction, a difference analysis module (DAM) is used to reveal discriminative stages on the surface of the lesion compared to the skin around the lesion. Finally, the Edge Guidance Module (EGM) combines these features with a database of edge structures and creates a fine edge map, which is then used to accurately segment the lesion.

#### Multilevel feature extraction

LEDNet utilizes the Siamese network to extract multi-level features from each pair of skin lesion images. The two branches are common and give the paired images into a common feature space. These features help this model capture the complex differences between the images. LEDNet is built on the U-Net architecture, which extracts features at many levels, including both a high-level semantic feature and a low-level spatial feature that is extremely important for the accurate boundaries of lesions. The feature extraction process is mathematically formulated as follows:

1 $$\begin{aligned} F_{p,q} = \psi _{p,q}(I_{p}, I_{q}), \quad p, q = 1, 2, \dots , N \end{aligned}$$

Where $F_{p,q}$ represents the features extracted from the $p^{\text {th}}$ and $q^{\text {th}}$ paired images $I_{p}$ and $I_{q}$ and $\psi _{p,q}$ denotes the function used to extract multi-scale features from the image pair.

#### Difference information extraction

The difference analysis module (DAM) improves feature differentiation between lesioned and non-lesioned regions through a proposed framework based on long-term short-term memory (LSTM). The DAM models temporal correlations between pairs of images using a “coarse-to-fine” approach, where the LSTM analyses similarities between features at different levels. A feature pair with a greater distance is assigned a higher weight, which means that the measures are more dissimilar. The module improves the model so that it is more robust and sensitive to the smallest changes in lesion boundaries despite noise or illumination changes. The DAM module can have the following output:

2 $$\begin{aligned} \tilde{D}_{p,q} = \text {LSTM}(F_{p,q}, W_{adjusted}) \end{aligned}$$

Where $\tilde{D}_{p,q}$ represents the difference information for the image pair $p, q$ and $W_{adjusted}$ denotes the dynamic weighting map that adapts to the feature variation.

The DAM can also be used effectively for the segmentation of skin lesions, as it captures the temporal dynamics of modelling feature-level changes in image regions. This enables finer differences between the edges of lesions and non-lesions to be detected even in low contrast or noisy backgrounds. Compared to conventional attention modules, DAM emphasises the clearly defined differentiation features, i.e., it does not focus on highly activated areas, but behaves more consistently when it comes to improving the boundaries of complex dermoscopic images.

#### Dynamic weight map

A dynamic weighting map, denoted as W adjusted, is an interactive attention block that has been integrated into the difference analysis module (DAM). It weighs more heavily the pixel pairs of the feature $(F_{p,q})$ that have larger differences, indicating possible boundaries of lesions. The weights are trained dynamically and this training is based on an LSTM network that captures the feature correlation between a paired lesion and non-lesion image. This method enables the network to highlight structural differences that are difficult to recognize on dermoscopic images.

#### Edge-guided change map estimation

Finally, in the last phase, an accurate edge map is calculated by the Edge Guidance Module (EGM), which consists of a mutual estimation of the change probabilities and the edge maps. The EGM consists of two branches, the edge estimation branch and the change map generation branch. The two branches generate probability maps of the edges and the lesion areas, which are optimized using a deep edge-driven loss value. The formulation of the loss is as follows:

3 $$\begin{aligned} \mathscr {L}_{EGM} = \alpha \cdot \mathscr {L}_{e} + \beta \cdot \mathscr {L}_{c} \end{aligned}$$

Where $\mathscr {L}_{e}$ and $\mathscr {L}_{c}$ represent the loss functions for edge estimation and change map generation, respectively and $\alpha$ and $\beta$ are the respective weighting factors that balance their contributions. The boundary map shows the location of the regional boundaries of the Swin-UMamba segmentation model.

EGM provides pixel-level boundary supervision by jointly estimating edge and change maps. This dual-branch design is more effective than traditional encoder-decoder skip connections because it introduces direct edge perception into the training objective. Attention blocks such as SE and CBAM assist with channel-wise relevance but do not guide boundary localisation. EGM addresses this by enforcing edge prediction during training, substantially improving segmentation around the irregular contours of lesions. The integration of DAM and EGM in LEDNet is motivated by the need to achieve accurate edge detection in dermoscopic image segmentation. Table 1 summarises the rationale behind the design of DAM and EGM and highlights their advantages over traditional methods such as SE blocks and skip connections. These architectural choices directly contribute to the improved segmentation performance observed in our experimental results.

**Table 1:** Comparison of DAM and EGM with traditional methods.

**Module**	**Function in LEDNet**	**Traditional methods**	**Limitation of traditional methods and justification**
Difference Analysis Module (DAM)	Enhances boundary differentiation by modelling spatial differences using temporal dynamics	SE Block, Attention Gates	These focus on channel-wise attention but lack explicit modelling of boundary variations. DAM improves DSC and boundary localization on dermoscopic images.
Edge Guidance Module (EGM)	generates edge and change maps for deep edge monitoring	Skip connections (U-Net), CBAM	these lack direct edge-oriented monitoring. EGM improves the sharpness of lesion boundaries and reduces the misclassifications of background pixels.

#### Deep edge-driven loss function

The Edge Guidance Module (EGM) generates two outputs: an edge probability map and a change probability map. To ensure the simultaneous training of both outputs, we use a deep edge-guided loss function that combines their respective binary cross-entropy losses. The total loss function is defined as:

4 $$\begin{aligned} L_{\text {EGM}} = \alpha \cdot \mathscr {L}_e + \beta \cdot \mathscr {L}_c \end{aligned}$$

where $\mathscr {L}_e$ represents the binary cross-entropy loss for the edge map, $\mathscr {L}_c$ corresponds to the loss for the change map and $\alpha$ and $\beta$ are scalar hyperparameters that control the relative importance of each component. This formulation ensures that both structural edges and region transitions are accurately captured and penalized during training, leading to enhanced lesion boundary segmentation. Edge-guided loss formulation has been effectively applied in more recent work, such as Xing et al. (2024)^15^.

### Lesion segmentation using Swin-UMamba

The Swin-UMamba model segments lesions in various datasets post edge extraction. Swin-UMamba is an advanced model for medical image segmentation. It uses a Mamba encoder to encode long-range dependencies, to encode multi-scale features for complex texture of skin lesions such as pigment networks.

#### Mamba-based Encoder with Visual State Space (VSS) blocks

The encoder employs Visual State Space (VSS) blocks to track the dependencies of shapes at multiple levels. Per VSS block operation, apply a 2D Selective Scan (SS2D) on the feature map, with each block utilizing four directions. Operation is defined as follows.

5 $$\begin{aligned} H_{VSS} = SS2D(F, \theta _{scan}) \end{aligned}$$

where *F* is the feature map and $\theta _{scan}$ is a scan parameter for the selective scan of the feature map. The model generates a feature map that captures the fine-grained and overall structure of the image.

#### Down-sampling

The encoder only gradually degrades the spatial resolution over several stages. Such down-sampling is made possible by the patches or merged layers, through which the model still preserves important information while excluding the higher-level information. The down-sampling process is defined as follows:

6 $$\begin{aligned} H_{down} = \text {DownSample}(H) \end{aligned}$$

In this phase, lesions of different shapes and sizes are recorded, reducing costs while capturing the details of the most important features.

#### U-shaped decoder

The decoder also uses the U-Net structure, in which the connections between the encoder and decoder stages pass on information that is useful for reconstructing the shape of the lesion. Each up-sampling block of the decoder has residual convolutional layers and depth monitoring improves the progressive segmentation. The up-sampling operation is mathematically formulated as follows:

7 $$\begin{aligned} H_{up} = \text {UpSample}(H_{down}, \theta _{up}) \end{aligned}$$

For the model where $H_{FFM}=down\_sampling$ and $\theta _{up}$ is the up-sampling parameter. The final segmentation map is obtained by a segmentation head for each scale to smooth and sharpen the lesion boundaries.

### Evaluation and results

Furthermore, to evaluate the sensitivity of the proposed model, experiments were performed on the Ph$^2$, ISIC-2017 and ISIC-2018 datasets regarding edge detection and lesion segmentation using recognized metrics such as Intersection over Union (IoU), Dice coefficient and Boundary F1 score. The following equation describes the evaluation process:

8 $$\begin{aligned} IoU = \frac{|P \cap G|}{|P \cup G|} \end{aligned}$$

Where $P$ and $G$ are the regions predicted by the model and the actual regions, respectively.

### Edge detection workflow

**Fig. 2:**
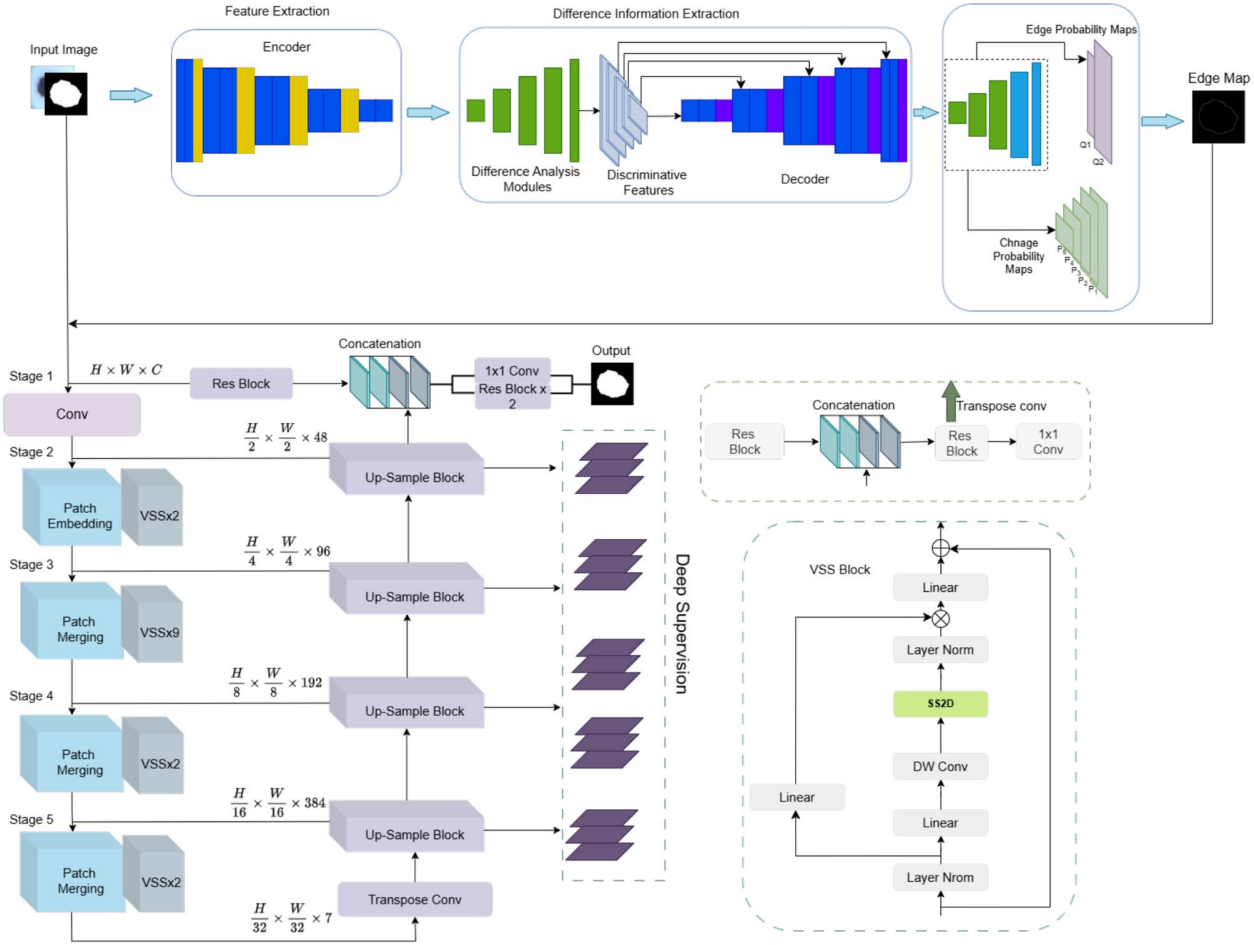
LEDNet architecture for efficient lesion detection.

The working of the edge detection process with LEDNet is shown in Fig. 2. The workflow includes the following steps: 1) Loading input and output image and mask. 2) Canny on the lesion mask. 3) Saving the edge image in the target directory.

The flow of the dataset through the proposed edge detection model is shown in Fig. 2. It starts with the import of the images and the masks for the lesions. The Canny edge detector is then used to detect the edges of the lesion masks. The generated edge maps are saved in a specific output directory. The integration of LEDNET and Swin-UMamba enables high-precision edge detection of lesions and segmentation that improves skin cancer diagnosis.

### Dataset

To validate the proposed LEDNet-UMamba model, we performed extensive experiments using three publicly available datasets to analyze dermatological lesions: ISIC-2017^42^, ISIC-2018^43^ and Ph$^2$^44^. ISIC-2017 and ISIC-2018 are benchmarks for the segmentation and classification of skin lesions collected by the International Skin Imaging Collaboration. The relatively limited Ph$^2$ dataset contains high-quality dermoscopic images that are ideal for external validation. This enables the evaluation of the model’s performance after training with the ISIC datasets.

Dermatoscopic images with segmentation masks were used for the ISIC 2017 dataset. The dataset was divided into 1400 images for training and 200 images for validation and the remaining 400 images were used for testing. All images used in this study were reduced from their original size of 576$\times$767 pixels to a standard size of 256$\times$256 pixels. The images of ISIC 2018 dataset is a collection of 2,594 images with segmentation masks. It consists of 1,816 for training, 259 for validation. And 519 for testing. The input image size for all the images also had to be standardised at 256 $\times$ 256 pixels.

Only external tests utilized the Ph$^2$ dataset with their associated segmentation labels. Such steps ensured the model’s stability and its ability to predict on fresh data without evaluation bias. The dimensions of the Ph$^2$ images were downscaled from 768$\times$560 pixels to 256$\times$256 pixels to fit the input size of the model. Making the input size uniform across all models assisted with dependable feature extraction and made the model generally usable. The proposed model was trained on the ISIC 2017 and ISIC 2018 and was evaluated on the Ph2 dataset in order to validate its capability to segment skin lesion images of varying datasets and resolutions. Combining these datasets also provided varied imaging conditions and annotation types, allowing a realistic evaluation of the model’s generalisation. A standardised training and assessment environment was applied to all datasets to incorporate different dermatological scenarios.

### Algorithm for proposed model

The procedure for edge detection and segmentation of lesions, as shown in Algorithm 1, begins with loading the image data set $I_i$ and the corresponding masks $M_i$. The pairs are then divided into training and test data sets. The masks $M_i$ are processed using the Canny algorithm to produce the edge maps $E_i$, which are stored in the output directory. The Swin-UMamba model takes both the image $I_i$ and the edge map $E_i$ as input and outputs the segmented lesions $S_i$.

To evaluate the performance of the proposed model, the Intersection over Union (IoU) and the Dice coefficient are used, with both metrics calculated for each test sample. These edge maps and segmented lesions are stored for future computational analysis.. This enables the detection and accurate segmentation of the skin lesions.

**Algorithm 1:**
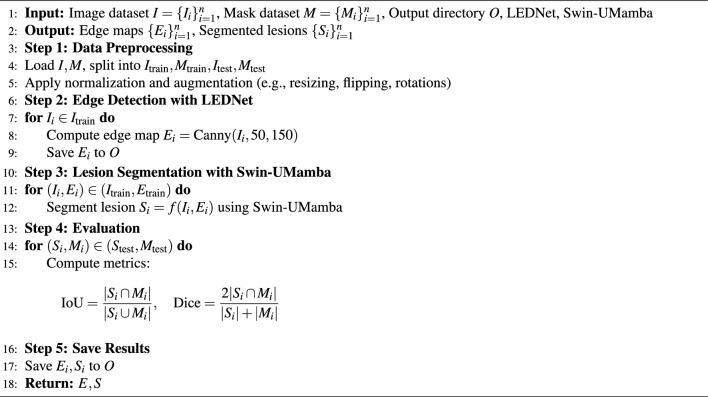
Edge Detection and Lesion Segmentation using LEDNet and Swin-UMamba

### Experimental setup

This section describes the experimental setup and the configurations used for this study. Hardware: to improve and speed up training with large images, the models were trained with an NVIDIA CUDA-compatible GPU to handle matrix operations and efficient backpropagation. Software: Image processing and modelling were implemented in Python, with image processing performed using OpenCV. LEDNet was trained with TensorFlow/Keras for edge detection and Swin-UMamba for lesion segmentation. Scikit-learn was used to generate the evaluation metrics, including the IoU and Dice coefficients, to analyze the performance of the model and the segmentation results were plotted using Matplotlib. The entire process of all models for training and evaluation was implemented on an NVIDIA CUDA-compatible GPU for computation. Table 2 provides the detailed distribution of training, validation and testing splits across the datasets used in experiments.

**Table 2:** Dataset distribution used in experiments.

**Dataset**	**Training**	**Validation**	**Testing**
ISIC-2017	1400	200	400
ISIC-2018	1816	259	519
Ph2	140	20	40

## Evaluation metrics

The performance of the lesion segmentation model is evaluated in this study using the following evaluation metrics: Dice Similarity Coefficient (DSC), sensitivity, specificity and accuracy. These are most commonly applied in medical image segmentation to measure the effectiveness of a segmented result. Dice Similarity Coefficient (DSC): Jaccard is applied to calculate the similarity between the true and predicted mask of the lesion. The evaluation of the segmented areas is especially important and a similarity close to 1 is considered a better match between the predicted and true areas of the lesions. The DSC is calculated as follows:

9 $$\begin{aligned} \text {DSC} = \frac{2 \times TP}{2 \times TP + FP + FN} \end{aligned}$$

where: $TP$ is the number of true positives, $FP$ is the number of false positives, $FN$ is the number of false negatives.

Accuracy (ACC) measures the percentage of correctly classified pixels (lesion and non-lesion). It is defined as:

10 $$\begin{aligned} \text {ACC} = \frac{TP + TN}{TP + TN + FP + FN} \end{aligned}$$

where: $TN$ is the number of true negatives.

Sensitivity (SE) measures the proportion of actual lesions correctly identified by the model. It is given by:

11 $$\begin{aligned} \text {SE} = \frac{TP}{TP + FN} \end{aligned}$$

where $TP$ is the number of true positives and $FN$ is the number of false negatives.

Specificity (SP) quantifies the model’s ability to identify non-lesion pixels correctly. It is calculated as:

12 $$\begin{aligned} \text {SP} = \frac{TN}{TN + FP} \end{aligned}$$

where $FP$ is the number of false positives and $TN$ is the number of true negatives.

Taken together, these metrics assess the performance of the segmentation model and indicate necessary improvements. The model can be used to identify the premorbid features of an individual.

**Fig. 3:**
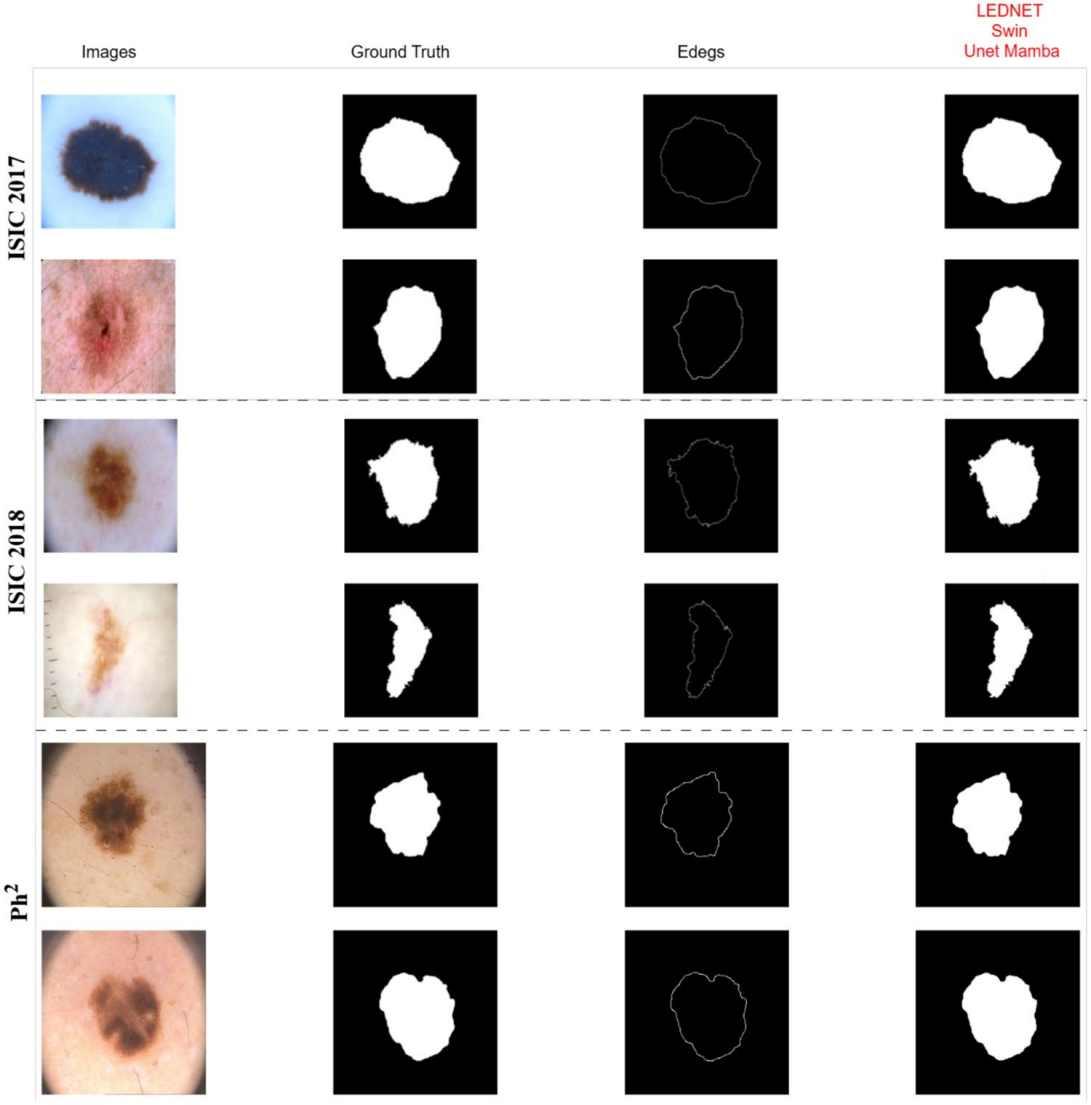
Comparison of the segmentation graphs with three publicly available data sets for skin lesion segmentation of skin lesions.

Figure 3 shows the segmentation model’s performance alongside the ground truth. The true positive areas in these outcomes indicate the correct identification of lesion regions. False positive areas occur where the model incorrectly predicts non-lesion areas as lesions, while false negative areas refer to lesion regions not identified by the model. The clarity of this assessment enables a definitive evaluation of the models’ capabilities in detecting the boundaries and shapes of lesions through a comparative visualisation of their segmentation results. It shows the overlap between the predicted lesion regions and the ground truth, as well as how effectively the model removes backgrounds from the segmentation.

By examining the image and calculating the above evaluation metrics, we can quantitatively evaluate the performance of the lesion segmentation model and ensure high precision in the identification and delineation of skin lesions.

## Experimental results and discussion

In this section, we analyse the performance of the proposed model using key metrics and compare it with the state of the art. This section describes the training and validation process and the classification measures used to evaluate the performance of the model. We compare these results with the conventional models to highlight the effectiveness and importance of the proposed model in the segmentation of skin lesions.

### Ablation study

The effects of the individual architecture components on performance in all three benchmark datasets are shown in Table 3. The integration of DAM and EGM consistently improves both overlap-based metrics (DSC, Jaccard), with the proposed model achieving the highest overall performance.

**Table 3:** Ablation study Comparison of the effects of the components of the three datasets.

**Configuration**	**DSC (ISIC-2017)**	**Jaccard (ISIC-2017)**	**DSC (ISIC-2018)**	**Jaccard (ISIC-2018)**	**DSC (Ph2)**	**Jaccard (Ph2)**
LEDNet only	0.845	0.762	0.862	0.782	0.912	0.856
Swin-UMamba only	0.861	0.781	0.878	0.801	0.918	0.865
LEDNet + Swin-UMamba	0.876	0.806	0.891	0.828	0.925	0.873
+ DAM	0.887	0.819	0.902	0.839	0.934	0.884
+ EGM	0.892	0.825	0.907	0.845	0.939	0.889
**Full Model (Proposed)**	**0.9734**	**0.9497**	**0.9753**	**0.9512**	**0.9801**	**0.9617**

### Comparison with existing methods

**Fig. 4:**
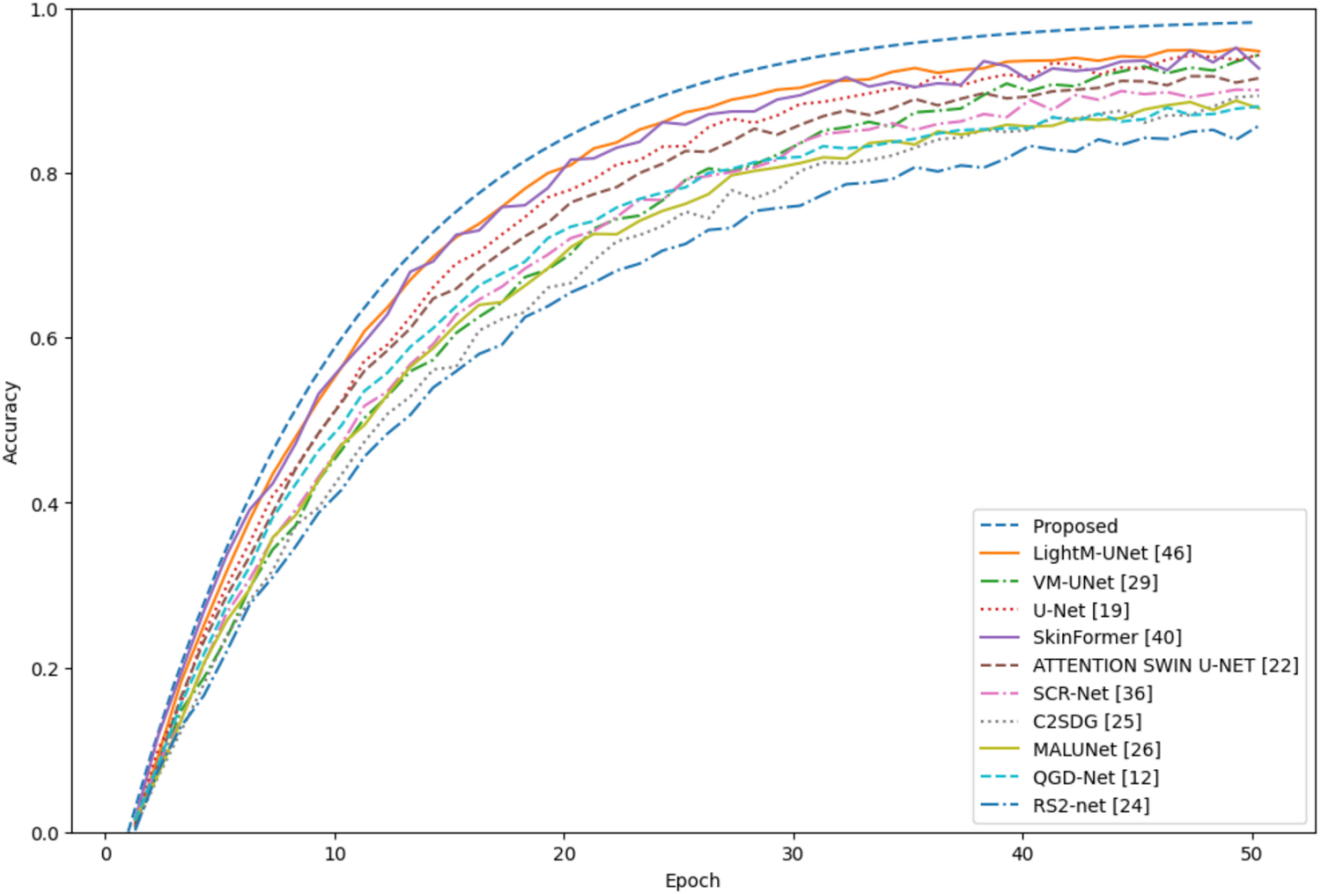
Comparison of the accuracy for the 2017 dataset.

The proposed model was evaluated with three different ISIC-2017 datasets, the ISIC-2018 dataset and the Ph$^2$ dataset to compare its performance with the state of the art. Each of the datasets was carefully selected to demonstrate the challenges and peculiarities of skin lesion segmentation and classification and to provide a comprehensive overview of the evaluation framework of the problem. ISIC-2017, ISIC-2018 and Ph$^2$ were the three benchmark datasets that were fully tested with the proposed model and its segmentation potential, generalization and robustness were determined. ISIC-2017 is a collection of labelled images of skin lesions, an important benchmark for dermatological image interpretation. Figure 4 shows, the proposed model outperforms state-of-the-art models in the areas of segmentation accuracy and overall performance during the training epochs over the LightM-UNet structures. It is able to achieve such good results because it can represent complicated spatial interactions and perform successful feature integration, which can contribute to accurate segmentation of the lesion even in difficult cases with odd boundaries or increased variability.

**Fig. 5:**
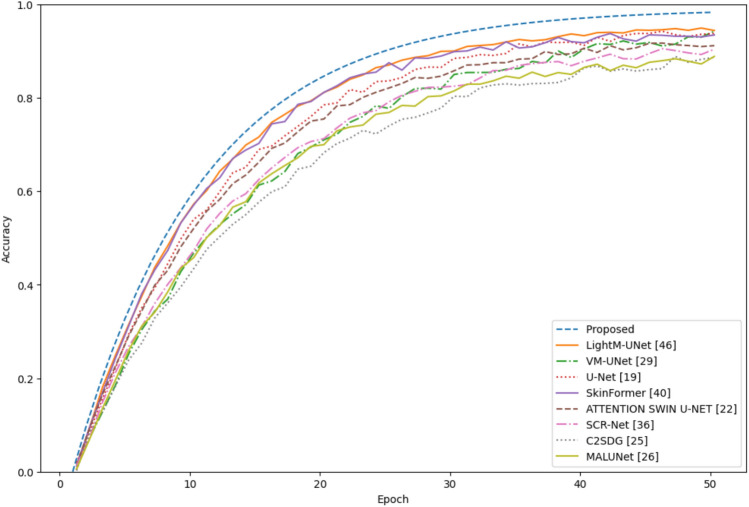
Comparison of the accuracy for the 2018 dataset.

To measure scalability, the ISIC-2018 dataset was used, which contains a larger set of lesion images. The results, shown in Fig. 5, emphasise the reliability of the model. It also showed better training and validation accuracy compared to other complex advanced architectures such as VM-UNet, Attention-Swin U-Net and SCR-Net. The loss curves show efficient optimization with fast convergence and stabilization at significantly lower values than competing methods, highlighting the adaptability of the model to the increasing complexity of the dataset.

**Fig. 6:**
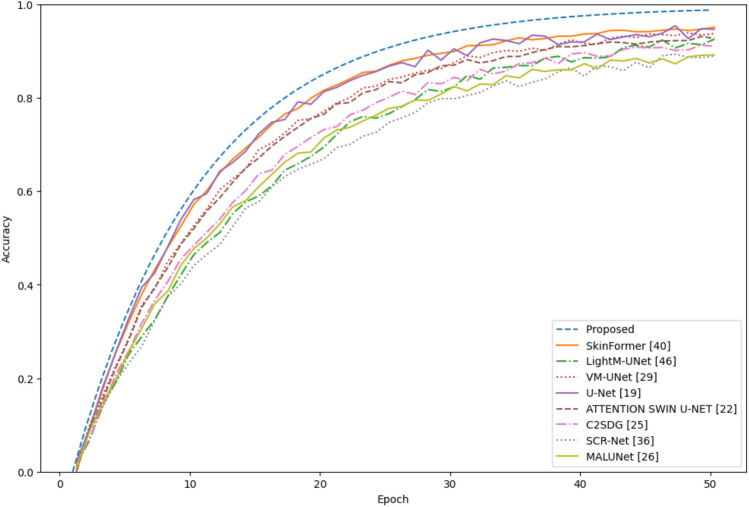
Comparison of the accuracy for the Ph$^2$ dataset.

The Ph$^2$ dataset, a smaller but high-resolution collection of melanoma images with detailed annotations, was used to test the model’s ability to perform accurate segmentation. The Ph$^2$ dataset has a smaller number of samples compared to the larger ISIC datasets, but the detailed annotations are required because the lesion boundaries need to be accurately identified. The results shown in Fig. 6 demonstrate the compatibility of the model with high-quality dermatological images and the ability to handle complex situations in contrast to other models.

The comparison with state-of-the-art (SOTA), such as SkinFormer^39^, LightM-UNet^45^, VM-UNet^28^ and MALUNet^25^ on the ISIC-2017, ISIC-2018 and Ph$^2$ datasets shows that the other systems manage specific tasks but struggle with complex spatial relationships. The proposed model, which consists of feature fusion and attention mechanisms, provides effective approaches to overcome these limitations and significantly improves performance. By combining datasets of varying complexity, resolution and labelling, this work has demonstrated the robustness and scalability of the proposed approach, which is effective in dermatological image analysis. The ability to process different data sets with minimal performance degradation is the potential of the method for real-world clinical applications where heterogeneity and variability of data pose a major challenge. Overall, these results demonstrate the effectiveness and performance advantage of the proposed model in overcoming the main challenges of skin lesion segmentation.

**Table 4:** Results of Comparison Experiments on the ISIC-2017 Dataset.

**Methods**	**DSC**	**SE**	**SP**	**ACC**
RS$^2$-net^23^	–	0.6580	0.8610	0.8870
QGD-Net^46^	–	–	–	0.9301
MALUNet^25^	0.8896	0.8824	0.9762	0.9583
SCR-Net^35^	0.8898	0.8497	0.9853	0.9588
C$^2$SDG^24^	0.8938	0.8859	0.9765	0.9588
ATTENTION SWIN U-NET^21^	0.8859	0.8492	0.9847	0.9591
SkinFormer^39^	–	–	–	0.9610
U-Net^18^	0.8989	0.8793	0.9812	0.9613
VM-UNet^28^	0.9070	0.8837	0.9842	0.9645
LightM-UNet^45^	0.9080	0.8839	0.9846	0.9649
**Current Study**	**0.9734**	**0.9697**	**0.9858**	**0.9847**

**Table 5:** Results of Comparison Experiments on the ISIC-2018 Dataset.

**Methods**	**DSC**	**SE**	**SP**	**ACC**
MALUNet^25^	0.8896	0.8824	0.9762	0.9583
SCR-Net^35^	0.8898	0.8497	0.9853	0.9588
C$^2$SDG^24^	0.8938	0.8859	0.9765	0.9588
ATTENTION SWIN U-NET^21^	0.8859	0.8492	0.9847	0.9591
U-Net^18^	0.8989	0.8793	0.9812	0.9613
VM-UNet^28^	0.9070	0.8837	0.9842	0.9645
LightM-UNet^45^	0.9080	0.8839	0.9846	0.9649
SkinFormer^39^	0.9320	–	–	–
**Current Study**	**0.9753**	**0.9494**	**0.9902**	**0.9713**

**Table 6:** Results of Comparison Experiments on the Ph$^2$ Dataset.

**Methods**	**DSC**	**SE**	**SP**	**ACC**
MALUNet^25^	0.8896	0.8824	0.9762	0.9583
SCR-Net^35^	0.8898	0.8497	0.9853	0.9588
C$^2$SDG^24^	0.8938	0.8859	0.9765	0.9588
ATTENTION SWIN U-NET^21^	0.8859	0.8492	0.9847	0.9591
U-Net^18^	0.8989	0.8793	0.9812	0.9613
VM-UNet^28^	0.9070	0.8837	0.9842	0.9645
LightM-UNet^45^	0.9080	0.8839	0.9846	0.9649
SkinFormer^39^	0.9270	–	–	–
**Current Study**	**0.9801**	**0.9892**	**0.9966**	**0.9932**

**Fig. 7:**
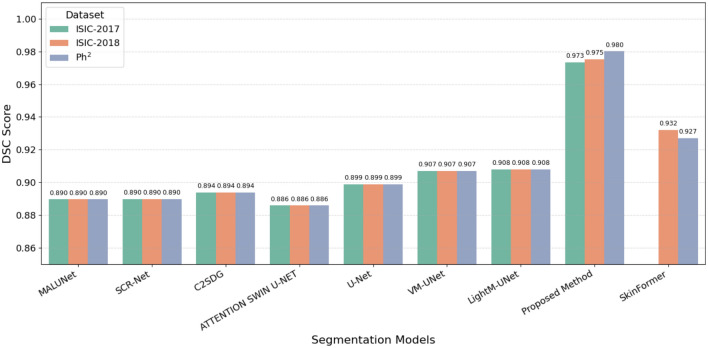
The proposed model’s DSC performance is evaluated across ISIC-2017, ISIC-2018 and Ph2 datasets.

A visual comparison of DSC scores across the three datasets is shown in Fig. 7, further illustrating the consistent superiority of the proposed method over baseline models.

**Fig. 8:**
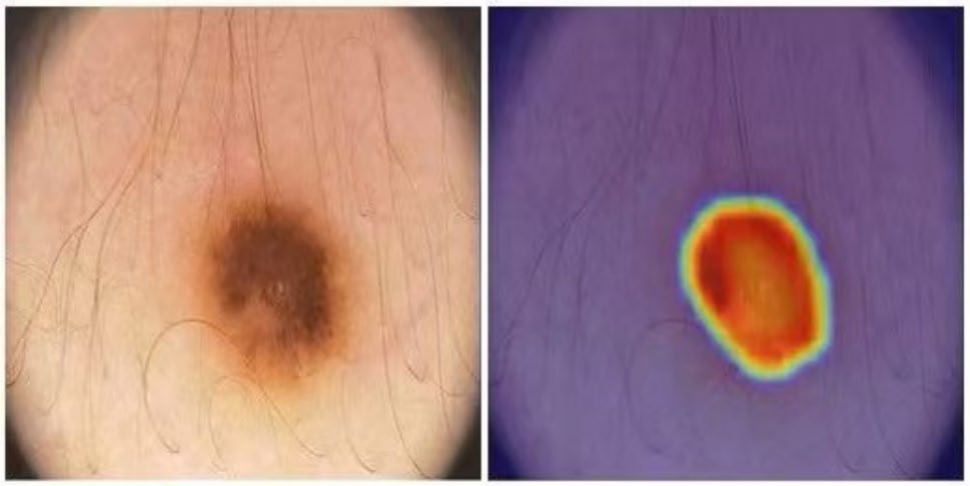
The heatmap (right) highlights the regions in the original dermoscopic image (left) that contribute most to the prediction of the model and shows a strong focus on the lesion area and its boundaries.

The interpretability of the model was examined by visualising the Grad-CAM attention maps. As shown in Fig. 8, attention is consistently focused on the lesion boundaries, the targets of the DAM and EGM modules and with clear clinical relevance.

## Discussion

The proposed model shows significant performance across multiple datasets. On ISIC-2017, ISIC-2018 and Ph$^2$, it achieves DSC values of 0.9734, 0.9753 and 0.9801, respectively, outperforming U-Net DSC = 0.8989 and SCR-Net DSC = 0.8898 tables 4, 5. The accuracy values of 0.9847, 0.9713 and 0.9932 also exceed the values of VM-UNet 0.9645 and MALUNet 0.9583. High specificity and sensitivity are achieved in particular with Ph$^2$ 0.9966 and 0.9892, as shown in Table 6. This is mainly due to Canny’s edge-based preprocessing, which highlights the lesion boundaries and complements the deep model. Compared to U-Net, SCR-Net and LightM-UNet, DSC = 0.8989, 0.8898, 0.9080, the proposed model achieves consistent performance improvements, especially on Ph$^2$, where high-resolution images and clear lesion boundaries match well with the edge guidance modulus. Datasets such as ISIC-2017, which contain noisy images and greater variability in annotations, show lower improvements, suggesting that the benefit of edge-aware modelling is partly data-dependent.

Although this study focuses on ISIC-2017, ISIC-2018 and Ph$^2$, future work will extend the evaluation to ISIC-2019 and ISIC-2020 to test robustness on larger and more heterogeneous datasets. Current limitations include performance degradation for highly occluded, low-contrast, or microscopic lesions where boundary labelling is weak. Approaches such as contrastive learning and multiscale refinement are being explored to mitigate these challenges. Variability between datasets can be explained by differences in sample size, resolution and quality of labeling. ISIC-2018 benefits from its larger scale, Ph$^2$ from high-resolution curated data, while ISIC-2017 presents more visually challenging cases with irregular lesion boundaries and low contrast. From a deployment perspective, the model is efficient and achieves real-time inference (approximately 25 FPS) on an RTX 3060 with memory consumption below 4 GB. The lightweight LEDNet decoder balances the heavier Swin-UMamba encoder and enables practical clinical integration. Model compression and optimizations (TensorRT, ONNX) are required for environments with limited resources. Further work is needed to ensure interoperability, interpretability and regulatory compliance.It is essential to use cross-data and data-independent validations, which are not included in this study, to test robustness under domain shifts. The model, which required on average about 38 ms for a single 512 $\times$ 512 image, demonstrates good real-time potential for clinical use. However, future research will examine quantization, pruning, and lightweight attention for mobile and edge applications.

## Conclusion and future work

This work proposes a hybrid model combining LEDNet and Swin-UMamba for robust skin lesion detection and segmentation. LEDNet employs a multi-level Siamese structure with a difference analysis module (DAM) and an edge guidance module (EGM) for efficient edge encoding. Swin-UMamba uses Mamba-based encoders and visual state space (VSS) blocks to capture long-range and contextual features. The integration of these components enhances segmentation, particularly for lesion boundaries and precise edge representation. The proposed method was evaluated using DSC, sensitivity (SE), specificity (SP), and accuracy (ACC) on three standard skin cancer datasets, consistently achieving significant performance. This technique may be valuable in clinical dermatology, as segmentation can assist in accurate diagnosis and treatment.

Future work will focus on optimizing the use of the system in real-time mode and applying it to other lesion types and data sets. Key directions include compressing the model through pruning and quantization, using ONNX or TensorRT for edge devices and exploring lighter attention mechanisms to replace the Swin-UMamba encoder. We also intend to incorporate neural architecture search (NAS) to find latency-optimized use cases that optimize segmentation accuracy with minimal computational effort. To further improve robustness and generalization, advanced post-processing tools and deep learning extensions are being explored.

## Data Availability

ISIC-2017 and ISIC-2018 were obtained from the ISIC Archive and are described in Gutman et al. (2016) and Codella et al. (2019), respectively, while the $\textrm{Ph}^{2}$ dataset was obtained from the INESC TEC Repository and is described in Mendonça et al. (2013). Derived data supporting the findings of this study are available from the corresponding author upon reasonable request.

## Code availability

The implementation of the hybrid LEDNet–Swin-UMamba segmentation framework, including training and evaluation scripts, is publicly available at https://github.com/asim-lab/Hybrid-LEDNet-SwinUMamba.
